# Rock Reinforcement by Stepwise Injection of Two-Component Silicate Resin

**DOI:** 10.3390/polym14235251

**Published:** 2022-12-01

**Authors:** Tatiana Shilova, Aleksander Serdyukov, Sergey Serdyukov, Oksana Ivanova

**Affiliations:** 1 Chinakal Institute of Mining, Siberian Branch of Russian Academy of Sciences, Krasnyi ave. 54, Novosibirsk 630091, Russia; 2 Department of Geology and Geophysics, Novosibirsk State University, Novosibirsk 630090, Russia

**Keywords:** rock reinforcement, stepwise injection, extrusion of solutions by gas, sand rock, two–component silicate resin, strength properties, structure

## Abstract

Our research aims to improve the efficiency of the reinforcement of loose rocks with two-component polymer resins. The standard approach consists of the injection of two pre-mixed components into a rock massive. We propose a stepwise injection of individual components of a resin into the rock and deep extrusion of the solutions into the rock by gas between the injection stages. The experimental results indicate that the proposed method provides a reduction of polymer consumption per unit volume of the rock, and an increase in the impregnation depth, area of the resin impact, and the reinforced rock volume in comparison with the conventional method of prepared resin solution injection. The cured resin partially fills the sand rock pore space, binds the grains, and acts as a reinforcing frame. The highest reinforcement is achieved with the sequential stepwise injection of the resin by separate small portions of each component. We have shown the uniaxial compressive strength is on average more than twice as high that obtained with the conventional injection method. This can be explained by higher fracture toughness of the reinforced rock with a flexible hardened network of the cured resin in the structure.

## 1. Introduction

We consider a method of rock reinforcement by two-component synthetic polymer resins. Synthetic polymer resins are widely used to reinforce rocks and soils. Our two-component polymer resin reinforcement consists of the injection of two pre-mixed components into a rock massive at the required depth. The components are mixed inside hydraulic system of injection equipment and pumped through pre-formed holes. The injection sites are selected according to rock type, groundwater level, etc. [1,2,3,4,5]. A thorough mixing of the resin components is crucial. A chemical reaction of the two mixed components inside a rock causes a cure of injected liquid polymer resin solution [6,7,8,9]. The advantages of the two-component resin reinforcement method are the rock stabilization under high loads and movements, performance, and the cure time control by varying catalyst content, etc. An effectiveness of the injection reinforcement is determined by radius/depth of an impregnation, which is directly affected by the path of resin solution penetration. During the injection the resin solution should penetrate through the rock pores, distributing a homogeneous mixture over the pore space [10,11,12,13]. The loose rock impregnation depth and the injected composition distribution depend on many factors, such as rock type, hydraulic conductivity, density, particle size distribution, injection pressure, dynamic viscosity of the resin solution, temperature of the mixed components, injected resin amount, etc. [14,15,16]. Let us observe some major phenomena and the related research of the considered method of rock reinforcement by resin injection.

The particle size of granular formations and rock permeability influence the distribution of the resin solution around the injection point. To maintain the composition flow rate as the permeability decreases, it is sufficient to increase the injection pressure. However, a high injection pressure can lead to a hydraulic fracturing. New fractures are the additional channels and, therefore, increase the distance of the flow from the injection point. As a result, the resin solution consumption is increased without an improvement of the strength properties of the reinforced rock. To prevent hydraulic fracturing and obtain a satisfactory rock treatment it is necessary to use a relatively low injection pressure and low solution flow rate [17]. Laboratory tests are widely used to optimize and improve the procedure of resin injection [18,19]. The resin solution is pumped into a rock sample to measure the final strength and filtration properties of the reinforced rock. The injection procedure should be simulated during these studies so that it can be applied in natural conditions. There are several laboratory methods to treat loose rocks with two-component polymer resins. The simplest one consists of mechanical mixing of the rock sample with the polymer resin solution. The required quantity of loose rock (sand) and solution are prepared. To prevent the interaction of polymer resin solution with water, it is immediately added to the rock and mixed until a homogeneous mixture is formed. The obtained mixture is poured into the molds completely or in layers, compacting at each stage. The samples, which are made from these molds after the resin curing, are used for further laboratory testing. An advantage of this laboratory testing method is its simplicity. However, the considered experimental testing procedure does not provide the parameters of the polymer resin solution injection process [20,21]. The results of the injection reinforcement of fine, coarse sand with a two-component epoxy resin, sodium silicate, cement grouts at a constant solution consumption are given in [17,22,23]. Anagnostopoulos et al. (2004, 2005) consider one-shot and two-shot pumping of modified solutions into 10 cm sand columns [22,23]. Dependences of physical and mechanical properties on the distance from injection point were experimentally studied. The experimental procedure includes the pumping of two-component epoxy resin and sodium silicate solutions by one-shot or two-shot injection processes. In the one-shot process, a mixtures of epoxy resin and sodium silicate solutions of various ratios are prepared before injection. The one-shot injection tests were carried out with a constant flow rate of 0.1 l/s, and a low pressure about 100 kPa. In the two-shot process, epoxy resin and sodium silicate solutions are sequentially injected. To prevent the leaching out of the epoxy resin, an initial pumping pressure of the sodium silicate solution is set at 50 kPa and is increased during the experiment to keep the flow rate constant. After grouted sand is left for a day, the samples are made for compression, density, porosity tests, etc. The tests demonstrate that the compressive strength of the one-shot process samples is lower than those for the two-shot process. The polymer additives to cement slurries also significantly improve the physical and mechanical properties of the grouted sandy soil [22,23,24]. Compositions of the Rosil mixes, based on colloidal silica and inorganic salt, were laboratory tested to improve their reduction in permeability and consolidation properties. This type of mixes is capable of permeating fine soils. Laboratory tests of grouted dense medium-fine-grained sand with an up to 10% silt content are considered in [17]. Their injection is performed in one phase, regulating the curing by adjusting the dosage of the inorganic salt. Once injected in the ground, these mixes are cured into a stable material. A new multi-grouting technology was developed to make the use of low injection pressures and practical flow. This technology allows the simultaneous and selective injection of different layers in the same grout hole, with real-time control and monitoring of the grouting process. The Rosil mixes and the injection technology were tested and provided positive results both permeability reduction and improvement of the sand soil strength characteristics [17]. The effects of colloidal silica on silty sand with different amounts of silt are studied in [25]. The strength properties of stabilized samples under long-term static loading are determined. The results of the triaxial tests show an increase in the drained cohesion parameter in samples, while changing the drained internal friction angle in silty sands causes a decrease [25]. The behavior of silica sol as a permeation grout in hard rock is studied in a laboratory. Results showed that the initial strength of silica sol, a few kPa, increases over time, and the hydraulic conductivity ranges from 10^−10^ to 10^−11^ m/s, which can be compared to low permeable clays. It shows great potential for use as an injectable hydraulic barrier [26,27]. The operational parameters of a different nanosilica-improved soils are studied after curing. The results of the uniaxial compression tests, the indirect tensile tests, and the direct shear tests allow us to estimate the potential of colloidal nanosilica and on the differences between the chemicals [28,29]. A laboratory study of crushed shale rock grouting with a two-component polyurethane composition under pressure is presented in [30]. The study includes measuring of the grouting pressure in the pump inside the reservoir with rock, volume of solution and its distribution in the rock. Initial conditions that could affect ultrafine and acrylate grout application effectiveness are studied by [31] The initial conditions are soil grain size and in situ moisture content. A dependence of grouting time on sand water saturation is studied [31]. A new type of permeable polymer material for grouting anti-seepage reinforcement of dam slopes is proposed in [32]. The analysis of the safety factor and failure probability of different types of slope before and after reinforcement, shows that the safety factor of the slope can be greatly improved after the slope is reinforced with permeable polymer grouting, but as the slope height increases, the reinforcement effect decreases gradually. In general, for medium and low slopes, the improvement effect of safety factor can reach about 50% [32].

As can be seen from the above review, there are many ways to optimize the effectiveness of the reinforcement for a specific application by handling a number of parameters. We suggest a more general approach to improve the two-component polymer resin reinforcement method, namely a component-by-component stepwise injection into the rock instead of pre-mixed solution injection. Due the proposed stepwise injection scheme, the reaction between components and the curing of the polymer resin occurs directly in the rock volume, which happens to be more advantageous. The efficiency of resin polymerization depends on a mixing uniformity and a distribution of reagent molecules in the prepared composition volume. A heterogeneity of the mixture impairs the interaction of substances, especially for quick setting polymer resins. At the same time, the usage of quick setting two-component polymer resins for rock reinforcement is complicated by a high viscosity of their pre-polymers, a limited injection volume due to the quick cure time, an increased reactivity of individual components, etc. These factors lead to decrease of the rock impregnation volume and contamination of the injection equipment [12,30]. The proposed stepwise injection method eliminates these problems. We also further enhance our approach by extrusion of the individual components of polymer resin deep into the rock by nitrogen between the injection stages. In order to advocate our scheme, we perform a series of laboratory tests.

## 2. Materials and Methods

### 2.1. Materials

#### 2.1.1. Sand

**Figure 1:**
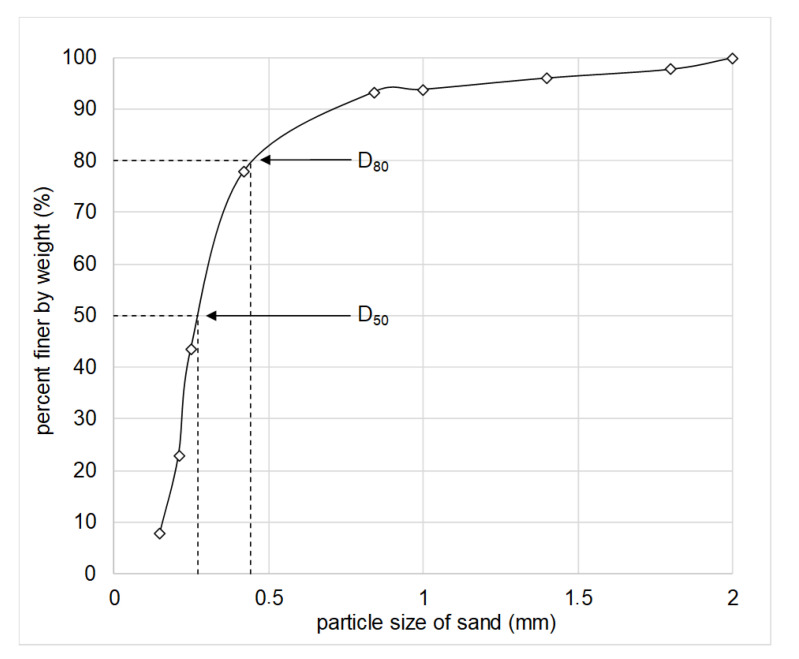
Particle size of sand.

Laboratory tests were carried out with pretreated fine sand samples. The sand was singled out near Novosibirsk city, Russia. The pretreatment includes a determination of the particle size distribution, absolute and bulk density, void ratio, etc. [33,34]. The sand is sieved through a set of sieves and the sand particle size distribution is determined. A portion of the sand with a particle size less than 0.42 mm is about 80 wt. % (D_80_, Figure 1). An average grain size is 0.27 mm (D_50_, Figure 1). The specific values of gravity and bulk density of the dry sand are 2.6 g/cm^3^ and 1.6 g/cm^3^, respectively. The void ratio is 0.66.

#### 2.1.2. Two-Component Silicate Resin

We use a non-expanding elastified two-component silicate resin in our experiments. The resin was designed to reinforce loose and disturbed rocks, to fill voids in a rock mass, to waterproof mines, etc. The two-component silicate resin is formed by mixing of two components (A and B) in a 1:1 volume ratio. The component A consists of sodium silicate solution (supplier is TEKC company, Saint Petersburg, Russia), distilled water, glycerol (supplier is Himreakt Co., Moscow region, Russia) and contains an addition of DMDEE (2,2-Dimorpholinodiethylether) catalyst (0.8 wt.%) (supplier is Haihang Industry Co., Ltd., Jinan City, China). The component B is a mixture of polymethylene polyphenyl isocyanate (supplier is Yantai Wanhua Polyurethans Co., Yantai City, China) and dibutyl phthalate (supplier is Himreakt Co.) (Table 1). Conventionally, mixed components A and B are injected into the rock in a volume ratio of 1:1. A part of the isocyanate reacts with water, which is contained in an aqueous sodium silicate solution of the component A. With the non-foaming elastified silicate isocyanate resin, the carbon dioxide is scavenged by sodium hydroxide forming soda and simultaneously causing the precipitation of silicon dioxide. The other part of the isocyanate reacts with each other, yielding a polyisocyanurate. Thus, both the silicate and the isocyanate form separate polymer networks, which penetrate each other. The thorough mixing of the two components is required to obtain an emulsion of sodium silicate in the isocyanate component and to form a homogeneous product [35]. An aqueous sodium silicate solution, being a strong alkali, interacts well with mineral grains, especially with carbonates and minerals of the gypsum group. As a result, the accelerated saturation of the reinforced rock with the resin solution occurs.

**Figure 2:**
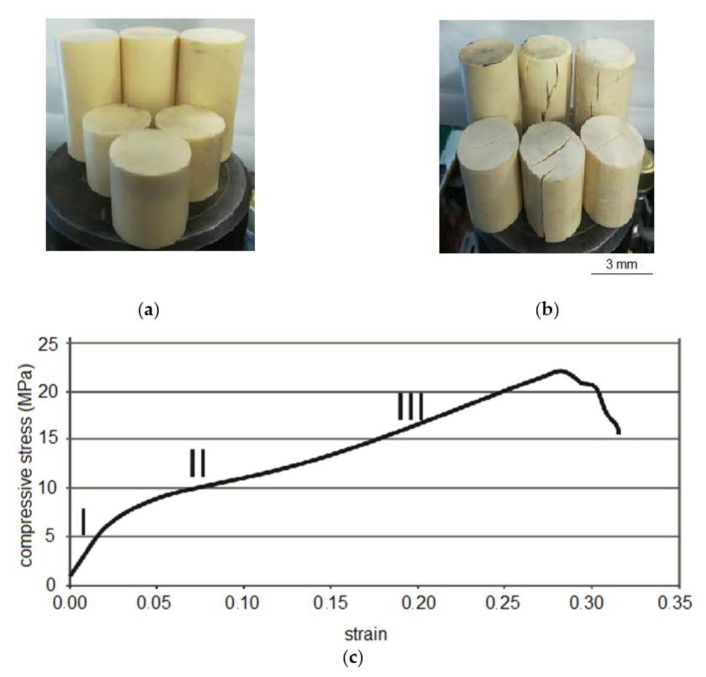
The two-component silicate resin: (**a**)—cylindrical specimens of the cured resin; (**b**)—the cured resin specimens after uniaxial compression tests with loading rate of 0.5 mm/min; (**c**)—stress-strain curve of the cured resin: I—linear section of the curve «stress-strain»; II—section, where stress increases slower; III—section, where stress increases faster until the uniaxial compressive strength of the sample.

The considered resin is applied in mining and underground building. Start and finish times of the resin polymerization (after mixing of components A and B) were experimentally established with a 0.8 wt.% catalyst content. The start and finish times are 135–150 s and 210–240 s at 25 °C, respectively. A foaming factor of the two-component silicate resin is equal to 1. Uniaxial compression tests of the cured resin were carried out under loading rate of 0.5 mm/min on cylindrical samples with a diameter of 37 mm and a length of 75 mm (Figure 2). An initial stage of the loading (strain is approximately 0.00–0.03) is characterized by a linear «stress-strain» curve (section I, Figure 2c). Then, as is typical for elastomers, the stress increases more slowly (strain is approximately 0.03–0.15; section II, Figure 2c) due to a breaking of bonds between macromolecules [37,38]. Then, the stress increases more quickly due to a reorientation of macromolecules depending on a direction of loading (section III, strain starts from 0.15, Figure 2c), and it continues until uniaxial compressive strength of the sample is reached. The uniaxial compressive strength of the cured two-component silicate resin is 20–23 MPa (Table 2).

### 2.2. Methods

**Figure 3:**
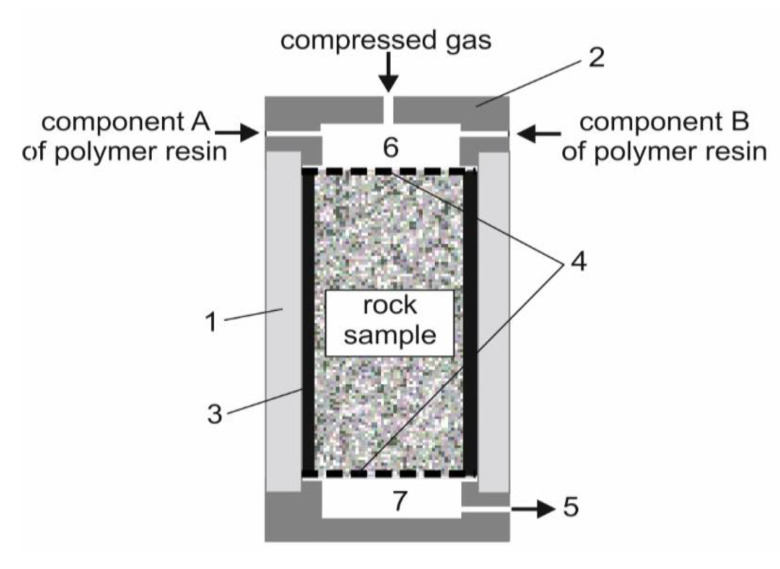
Test chamber of the laboratory stand to reinforce loose rocks: 1—metal case; 2—cover containing three inlets to pump solutions and compressed gas into a rock; 3—cylindrical shell; 4—perforated permeable plates; 5—outlet; 6—mixer; 7—receptacle.

We propose and experimentally test a method of sand rock reinforcement by a sequential separate stepwise injection of polymer resins. A laboratory stand was developed to carry out experiments. The stand consists of a test chamber, a two-channel hydraulic station to pump resin components, and a pneumatic system to extrude solutions with compressed gas into the rock. The test chamber is a metal case (1) with a cover (2), containing three inlets to connect injection hydraulic hoses and a compressed gas supply line. A replaceable plastic cylindrical shell (3) with an outer diameter of 10 cm and a wall thickness of 4 mm is placed in the case (1). A sand sample is placed inside a plastic shell (3). Its diameter is 9.2 cm, height is up to 30 cm. The ends of the sample are covered with perforated permeable plates (4), which form a flat resin filtration front and prevent the sand from escaping. In a bottom of the case there is an outlet (5) to drain remains of the solutions. The system of pumping the solutions into the rock samples provides both one-shot and two-shot injection of polymer solutions. During the one-shot injection, the components are pumped into a mixer (6) at the same time. A vortex mixing of the pumped solutions provides uniform distribution of their molecules in the prepared resin (Figure 3). During the two-shot injection each component of the two-component polymer resin solution is sequentially pumped. After the injection of each component, it is extruded into the rock with compressed nitrogen. The range of injection pressure is from 0.01 to 1.5 MPa.

**Figure 4:**
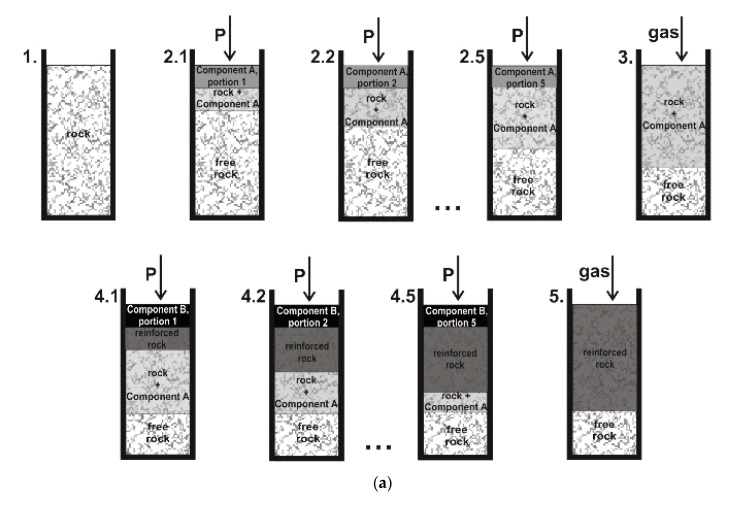

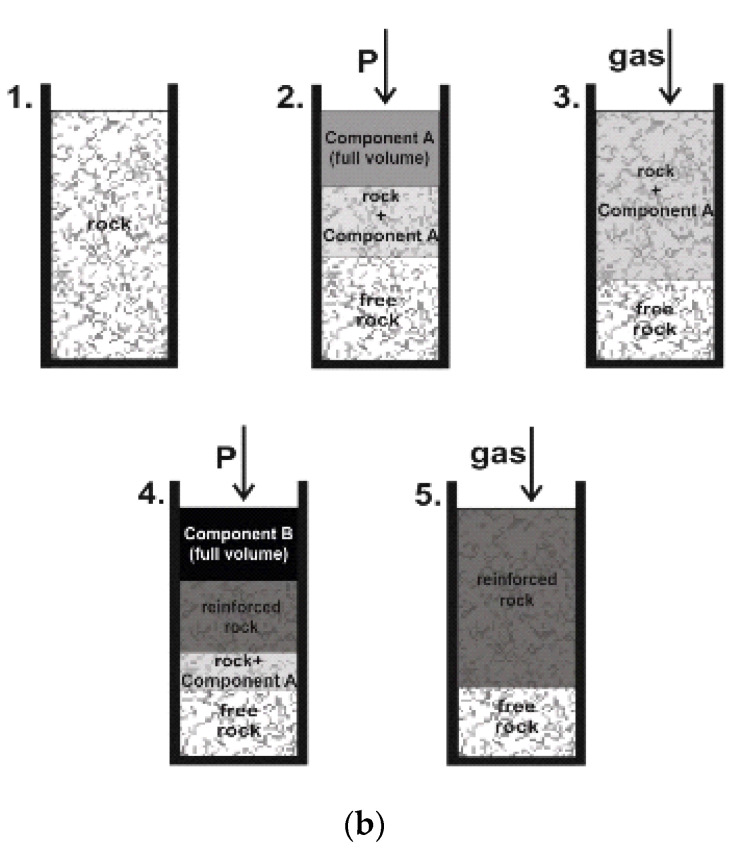
Sand rock reinforcement by the stepwise injection of the components A and B of the two-component silicate resin with their extrusion by gas into the rock between the injection stages: 1—a placement of the rock in the test chamber of the laboratory stand; 2—the injection of the full volume (**b**) or the small volume portions (**a**) of the component A, “P” stands for injection pressure. A number of substages (2.1, 2.2, …, 2.5) are representing the step-by-step injection of the component A portions (**a**); 3—the extrusion of the component A of the resin solution with the compressed gas (nitrogen); 4—the injection of the full volume (**b**) or the portions of the component B (**a**). A step-by-step injection (4.1, 4.2…4.5) of the component B portions (**a**); stage 5—the extrusion of the component B of the resin solution with nitrogen.

The experimental procedure of the stepwise injection includes several stages:

1. An installation of the replaceable cylindrical shell with a sand rock sample in the test chamber.
2. A preparation of the required volume of component A of the resin solution and its injection into the rock sample. The component A is pumped in two ways. The first one is the sequentially pumping of small portions of the component A. To increase the impregnation of the sand rock the system is kept under pressure after each injection stage (Figure 4a). The second pumping approach is the injection of the full volume of the component A into the rock (Figure 4b).
3. A pumping of the compressed nitrogen through the rock sample to extrude the injected component A deep into the rock (Figure 4a,b).
4. A preparation and an injection of the component B into the rock sample. The component B is also pumped in the same two ways as the component A (see p.2). The injection schemes are shown in Figure 4a,b.
5. A pumping of the compressed nitrogen through the rock sample to extrude the injected component B deep into the rock (Figure 4a,b).
6. A time delay, which is required for the complete polymerization and achieving of sufficient strength properties of the cured resin.

**Figure 5:**
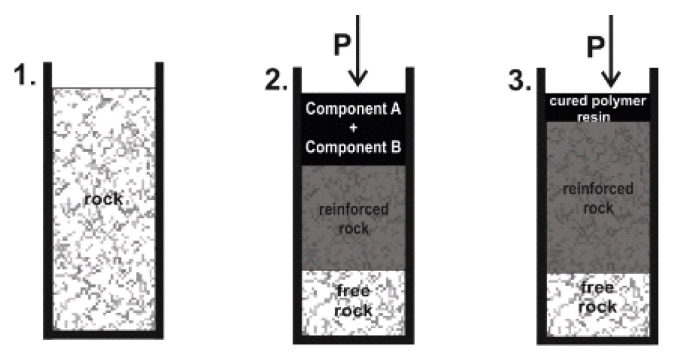
The sand rock reinforcement by stepwise injection of the prepared two-component silicate resin: 1—the installation of the rock in the test chamber; 2—the injection of the prepared resin solution (mixture of components A and B in a volume ratio of 1:1); 3—the extrusion of the injected resin solution with compressed nitrogen deep into the rock.

The injection of prepared two-component silicate resin solution has also been studied. The resin solution is pumped immediately after mixing components A and B in a 1:1 volume ratio. The experimental procedure includes several stages:

- An installation of the replaceable cylindrical shell with a rock sample in the test chamber.
- Mixing the equal volumes of the two-component silicate resin components A and B. The injection of the prepared resin solution into the rock sample with a time delay at the maximum value of the injection pressure (Figure 5).
- A time delay, which is required for the complete polymerization and achieving sufficient strength properties of the cured resin.

The total volume of the resin solution is 220 cm^3^ for all considered injection schemes, which is 1.4–1.5 times less than the pore volume of the sand rock sample. The injection pressure is 0.5 MPa. This is enough to pump the selected volume of resin components or the prepared resin. In the case of portioned injection, the volume of one portion of each component (A and B) is 20–22 mL. The time delay of the system under the pressure after injection of the next portion is 60 s. Both in the case of the stepwise portioned injection and the full volume injection of the A and B components a compressed nitrogen is pumped through the sample to extrude the resin components deep into the rock at a pressure of 0.5 MPa. The injection of the prepared resin solution lasts 10 min. The cylindrical shell with impregnated rock is left for 24 h. Then the reinforced rock is extracted for the laboratory tests. We estimate the volume of the reinforced rock and perform a microstructural analysis of the obtained samples.

We use scanning electron microscopy to determine the structural features of the reinforced sand rock samples. Samples are studied using a MIRA 3 LMU scanning electron microscope. We use this method to establish a relationship between the spatial distribution of sand grains and aggregates of the cured polymer resin. The sand grains, the cured polymer aggregates, structure heterogeneities, and voids, etc., are identified on the core scale [40]. In addition, the inter-granular distance is determined to estimate the compaction of bulk sand rock as a result of the resin injection. To characterize the filling of voids in the reinforced rock, a quantitative porosity estimation on basis of the obtained images fragments is performed [41].

We also perform deformation-strength tests of the reinforced sand. 3 cm diameter and length cylindrical samples are drilled out. They are used for uniaxial compression tests with an INSTRON 8802 servo-hydraulic press. We implement a speed-controlled traverse to measure and record the applied force and deformation [42,43]. All tests are made at a constant traverse speed of 0.5 mm/min. The samples are loaded up to their destruction. As a result, the uniaxial compressive strength is determined.

## 3. Results and Discussion

**Figure 6:**
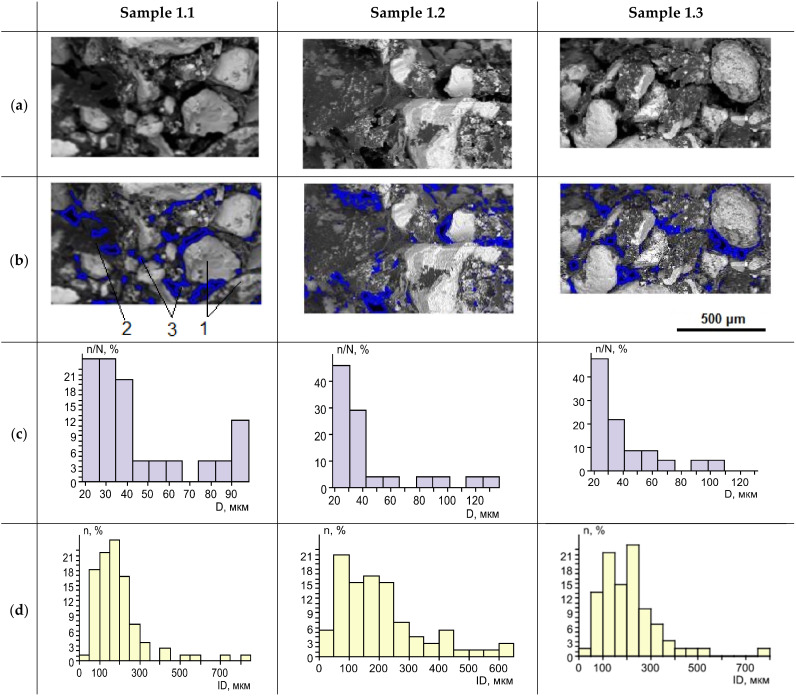
The structure of sand, reinforced by the stepwise injection of the two-component silicate resin. (**a**). Backscattered electron images of the samples; (**b**). the images with highlighted inter-grain voids: 1—sand grains; 2—aggregates of cured polymer resin; 3—inter-grain voids; (**c**). histogram of the void sizes (D—diameter) distribution; (**d**). distribution of the inter-void distances.

We consider two methods of two-component silicate resin rock reinforcement: the stepwise component-by-component injection, which is a novel approach, and the injection of a prepared two-component mixture. We study and compare the distributions of the cured resin in sand pore space. In the case of the stepwise injection with the extrusion of the components with compressed nitrogen deep into the rock, the cured resin forms films on mineral grains, which partially fill the pores. It happens both when the resin components are injected by portions and in the full volume. The films of the cured resin form aggregates, which provide an adhesion of sand grains, and form a reinforcing three-dimensional frame, which connects mineral grains into aggregates (Figure 6). We observed large inter-granular voids in the reinforced rock. Also, we observed small voids, which are localized in the cured silicate resin. An average size of the large inter-granular voids is 41 μm with an inter-void distance of 204 μm. The size of small pores is about 8 times smaller than the large ones (Figure 6). A residual porosity of the sand rock, reinforced by the stepwise injection of two-component silicate resin, is 5.8–12% with an average of 9%. An average distance between sand grains in the reinforced rock samples is about 245 µm.

**Figure 7:**
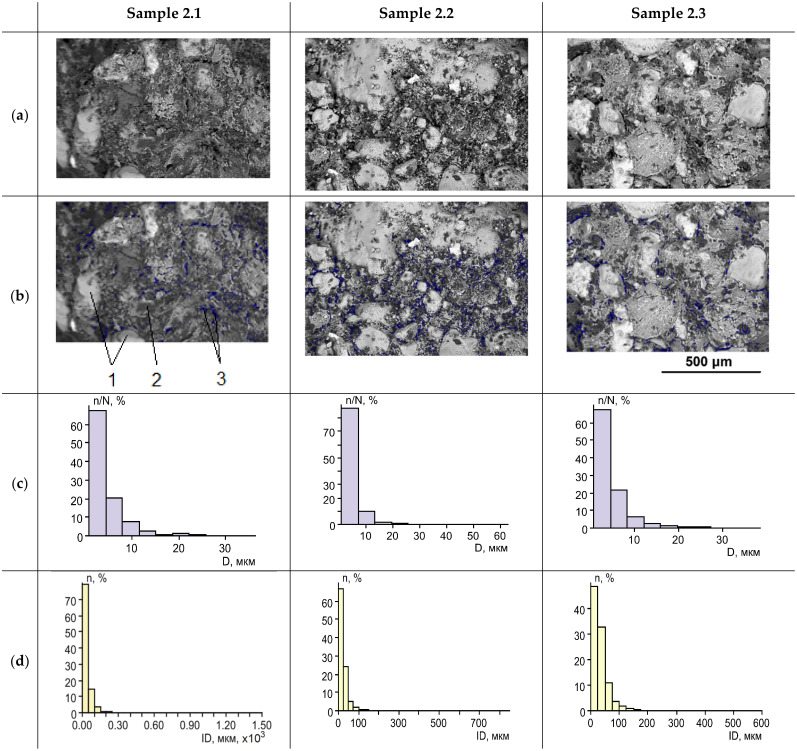
The structure of the sand reinforced by the injection of a prepared two-component silicate resin solution: (**a**). Backscattered electron images of the samples; (**b**). samples with highlighted inter-grain voids: 1—sand grains; 2—aggregates of cured polymer resin; 3—voids; (**c**). distribution histogram of the void sizes (D—diameter); (**d**). distribution of inter-void distances.

In the case of the sand reinforcement by prepared two-component silicate resin solution the structure of the obtained samples is more homogeneous. The cured polymer resin almost completely fills the pore space of the reinforced rock (Figure 7). Residual voids are small closed pores inside the cured resin and near the surface of the sand grains. The porosity is 2–4.5% with an average of 3.4%. The average void size is 5 µm with a distance between voids of 32 µm (Figure 7). The average distance between sand grains in 253 µm. It can be noted that the sizes of the closed voids inside the cured silicate resin for both considered methods of the resin injection are similar, with average values of 4–5 µm.

The stepwise injection of the two-component polymer silicate resin (both injection in portions and injection of the full volume of each component) with the compressed nitrogen driven extrusion provides 42% reinforcement of the sand sample. The injection of the prepared silicate resin provides 14% reinforcement of the sand sample. The stepwise injection increases the impregnation depth and the volume of the reinforced rock by a factor of approximately 3, compared with the injection of the prepared silicate resin. The average polymer consumption per unit volume of the reinforced sand rock is 0.37 with the stepwise injection and 0.29 with the injection of the prepared silicate resin solution. The lower penetration of the prepared resin is probably due an increase of its viscosity during polymerization after mixing the components. Dynamic viscosities of the unmixed components A and B are 137 mPa·s and 135 mPa·s respectively for temperature 25 °C [30]. The experimentally determined viscosity of the prepared polymer silicate resin is approximately 3000 mPas in 10 s after mixing the A and B components and increases up to 8400 mPas in 80 s.

**Figure 8:**
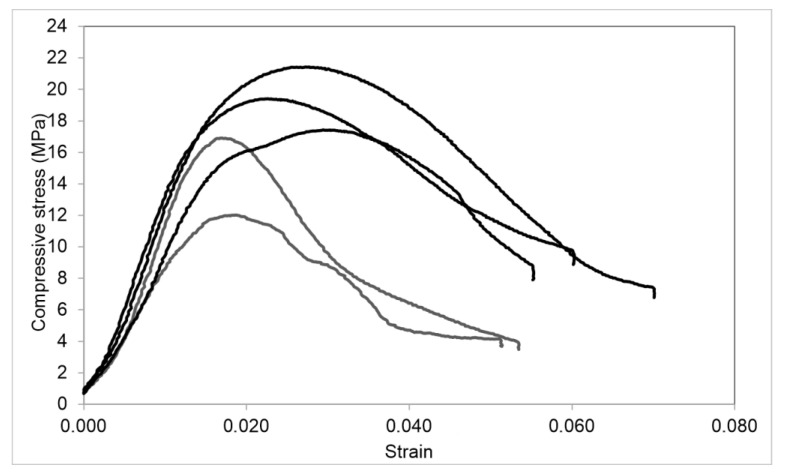
The results of uniaxial compression tests with loading rate of 0.5 mm/min: black curves—stress-strain diagrams for the sand reinforced by the sequential pumping of small volumes of components A and B with a retention of the system under pressure after each stage and their extrusion with the compressed nitrogen rock (the injection scheme is shown in Figure 4a); grey curves—stress-strain diagrams for the sand reinforced by the stepwise injection of the full volumes of components A and B into the rock and their extrusion by compressed nitrogen deep into the rock (the injection scheme is shown in Figure 4b).

**Figure 9:**
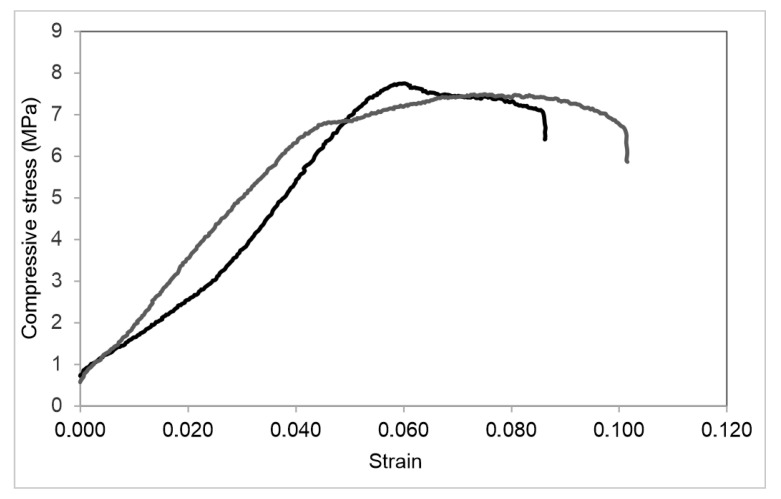
The results of uniaxial compression tests with loading rate of 0.5 mm/min: stress-strain diagrams for sand reinforced by the injection of a prepared two-component silicate resin solution.

The efficiency of the considered methods of the two-component silicate resin injection is estimated according to the strength tests of the reinforced sand samples. The results of the stepwise injection of the two-component silicate resin are shown in Figure 8. The results of the prepared two-component silicate resin injection are shown in Figure 9. The analysis of the obtained experimental data indicates that the stepwise injection of the two-component silicate resin with the extrusion of the components by compressed nitrogen provides the higher mechanical strength of the reinforced sand. The highest uniaxial compressive strengths are achieved by the sequential pumping of the small volumes of the components A and B with a retention of the system under the pressure after each stage, which increases the impregnation of the sand rock (Figure 8; the injection scheme is shown in Figure 4a). The average uniaxial compressive strength is 19,4 MPa, which is 1.6 times higher than the value, obtained for sand, reinforced with the stepwise injection of the full volumes of components A and B (Figure 8; the injection scheme is shown in Figure 4b).

The uniaxial compressive strength of sand, reinforced by the injection of the prepared two-component silicate resin solution, is on average 7.4 MPa, which is significantly lower than the values obtained for the sand, reinforced by a stepwise injection of a two-component polymer resin (Figure 9; the injection scheme is shown in Figure 5). At the same time, the sand impregnated with the prepared resin solution, withstands more significant longitudinal deformations. The uniaxial compressive strength is reached at strain 0.35–0.75, while the same parameter for the stepwise injection is reached at strain 0.18–0.25 (Figure 8 and Figure 9). The ascending curves of the stress-strain diagrams of the reinforced sand show that the stress increment increases from the certain load. We observe the similar stress-strain dependence for the cured two-component silicate resin, which has the properties of the elastomer. However, if the stress increment is noticeable from a stress of 6–8 MPa for the samples of the cured resin, then for the reinforced sand the increment begins at 1.5–3 MPa (Figure 8 and Figure 9). Thus, the reinforced rock exhibits the properties inherent in elastomers, but less than the cured polymer resin.

The performed deformation-strength tests demonstrate that the best sand reinforcement is achieved with the stepwise injection of the two-component silicate resin by small portions of each component, combined with their extrusion by compressed nitrogen. Compared with conventional methods, the proposed one provides a higher strength of the reinforced rock at lower specific consumption of the polymer resin per rock volume. This can be explained by the higher fracture toughness of the rock, reinforced with a flexible hardened framework, which is formed of thin films of the cured polymer resin mainly located on the surfaces of the rock grains that bind individual grains into consolidated rock aggregates. In case of the prepared solution injection, a more homogeneous, brittle structure with a continuous filling of the pore space with a polymer resin is observed. Thus, a higher crack resistance is achieved for the stepwise injection. The proposed stepwise sequential injection provides the reduction of the resin consumption per unit volume of the reinforced rock while improving its physical and mechanical properties.

## 4. Conclusions

We improve the method of the physical and chemical reinforcement of loose and disturbed rocks by the injection of the two-component polymer resins instead of the injection of two pre-mixed components, which is a conventional approach. We suggest the sequential stepwise injection of the two components of the polymer resin with the extrusion of each component by compressed gas deep into the rock. Due this injection scheme, the reaction between components and the curing of the polymer resin occurs directly in the rock volume, which is advantageous. 

We experimentally studied the proposed method and compared it with the conventional one. A series of laboratory tests on fine sand samples were performed and several results have been obtained:

- The reduction of polymer consumption per unit volume of the reinforced rock in comparison with the conventional method of prepared resin solution injection is observed. The increase of impregnation depth and the triple increase of the reinforced rock volume are observed.
- As seen in Figure 6, the cured polymer resin partially fills the sand pore space, binds the grains, and affects the reinforcing frame. As the result, large voids, located in the rock inter-granular space, are formed. Their size is on average 8 times larger than the small pores, which are located in the structure of the cured silicate resin;
- The higher strength properties at lower specific resin consumption per unit volume of the rock are observed. As seen in Figure 8 and Figure 9, the uniaxial compressive strength is on average more than 2 times higher than that obtained with the conventional injection method. The highest reinforcement efficiency is achieved with the sequential stepwise injection of the resin by separate small portions of each component. This can be explained by the higher fracture toughness of the reinforced rock with a flexible hardened network of the cured polymer resin compared to a more homogeneous structure, which is observed for the prepared resin solution injection. The cured polymer resin network probably prevents a formation of macro-cracks. The reinforced rock exhibits properties inherent in elastomers, but to a lesser extent than the cured polymer resin. These effects should be considered in further research.

## Figures and Tables

**Table 1:** Initial materials for silicate resin components.

Composition	Technical Document	Appearance	Properties
Sodium silicate solution	CAS 1344-09-8	Thick clear yellow or gray liquid	Silicate module 2.3–2.8; density at 20 °C 1.45–1.50 g/cm^3^
Glycerol	CAS 56-81-5	Odorless, viscous, clear, hygroscopic liquid	Glycerine content ≥ 99.5 wt. %; density at 20 °C ≥ 1.255 g/cm^3^; boiling temperature 290 °C
Distilled water	GOST R 58144-2018 [36]	Colorless liquid	-
DMDEE (2,2-Dimorpholinodiethylether)	CAS 6425-39-4	Yellowish liquid with an amine odor	Density at 25 °C 1.06 g/cm^3^; viscosity at 20 °C 29 mPas; boiling temperature 309 °C; ignition temperature 146 °C
Polymethylene polyphenyl isocyanate (Wannate PM-200)	CAS 9016-87-9	Dark brown thick liquid	NCO content: 30.0–30.2 %; viscosity at 25 °C 200–250 mPas; density at 25 °C 1.22–1.25 g/cm^3^
Dibutyl phthalate	CAS 84-74-2	Clear liquid without mechanical impurities	Density at 20 °C 1.045–1.049 g/cm^3^; flash point 168 °C

**Table 2:** Properties of two-component silicate resin [39].

Parameter	Value
The volume ratio of components A and B for mixing	1:1
Time of the start of the polymerization reaction, s	135–150
Time of the complete curing, s	210–240
Foam factor	1
Temperature (max) of reaction, °C	≤75
Density at 25C (Component A/Component B), g/cm^3^	1.38/1.16
Viscosity at 25C (Component A/Component B), mPa·s	137 ± 2/131 ± 5
Uniaxial compressive strength, MPa	20–23

## Data Availability

The data presented in this study are available on request from the corresponding author.
